# Mercy Hospital

**Published:** 1879-08

**Authors:** E. C. Helm

**Affiliations:** House Surgeon


### ARTICLE VII.

### MERCY HOSPITAL.

Reported by Dr. E. C. HELM, House Surgeon.

*I. Obstinate Ulcer of Foot.*

D. S., aged 34, entered the hospital March 21, 1879, with the following history :

Three years ago his left foot was crushed between two logs. Part of the heel and nearly all of the superficial tissues on the under and inner sides of the foot were torn away, leaving a large rectangular ulcer. This improved rapidly until the ulcer was about .08 m. square.

In spite of all treatment (including skin-grafting by a Canadian surgeon), this refused to heal.

At date of admission the edges of the ulcer were greatly thickened and indurated and the floor non-granulating. There was very little discharge, the ulcer being exceedingly indolent. No bones are known to have been broken — still the foot is flat. He is a very muscular, apparently healthy man, but is a victim of periodical methomania.

The edges of the ulcer were pencilled with tincture of iodine, and the whole covered with simple cerate. The internal treatment was 2 Gms. of sulphur three times a day.

After two weeks (April 2d), the ulcer meantime not improving, the iodine was discontinued, and firm strapping tried.

April 6th–9th.—Upon returning from a debauch, the foot was worse than ever.

April 10th. — Scarified edges and base of ulcer freely.

April 11th–13th. — Applied tincture of cantharides twice daily with camel's hair pencil.

April 14th. — Ether was administered, the edges of the ulcer were pared, the base scraped, several pieces of skin from the posterior surface of the leg were engrafted and the ulcer dressed with simple cerate.

After forty-eight hours, the dressings were carefully removed ; only three grafts remained, and these came away twenty-four hours later.

April 23d.—Scarified and grafted again.

April 25th–27th.—Another debauch. Grafts failed. Sulphur discontinued.

May 5th.—Anæsthetized, scarified parts well, and dissected up three large flaps extending from the edges of the ulcer well into the healthy tissues. These flaps were on the anterior, superior and posterior borders of the ulcer. They were left with broad attached bases, and were not twisted or stitched, as their vitality was not assured; but were dressed with simple cerate and bandaged loosely.

May 8th.—All of the anterior and posterior and part of the superior flaps have sloughed.

May 9th.—Directed potassium iodide 5 Gms. three times a day.

May 12th–16th.—Applied cantharidal blisters twice per day, leaving them on continuously. The blisters caused some pain, discomfort and an increased vascularity of the parts.

May 17th–22d.—Dusted on thickly a coating of capsicum twice per day.

May 22d.—Scarified again and continued the capsicum until June 1, at which time there was more redness, and a better crop of granulations than at any time before. Consequently the house-surgeon engrafted fourteen pieces of skin from the forearm, and dressed lightly, leaving the dressings undisturbed for forty-eight hours, at which time the grafts were all in position.

June 5th.—The grafting failed. The ulcer is healing rapidly where the flaps made May 5th were situated, while the original ulcer has changed but little since entering, except that it is very slowly granulating at the base, while the edges are still indolent.

June 6th.—Becoming discouraged he has left the hospital.

*II. Epithelioma.*

A. N., aged 55, entered the hospital April 9th, with an epithelioma of lower lip, of two years' duration. He could assign no cause for it other than that he was an inveterate smoker, always using a clay pipe. He had endured some pain of a smarting, rather than a shooting character. Last winter the lip was partially destroyed by caustics which were applied by a "cancer doctor."

April 14th.—Ether was administered and a free incision made, cutting out a quadrilateral piece of the lip, taking away fully three-fourths of its substance. The moderate hæmorrhage was readily controlled by the ligature and torsion. Next incisions .03 m. in extent were made from each lower angle of the quadrangle horizontally outwards. Like incisions were made from each angle of the mouth directly outwards, and thus parallel to the lower incisions. The flaps thus formed were separated from the periosteum, brought carefully into apposition, stitched and firmly strapped. There was considerable oozing for two days.

April 21st.—Removed the sutures.

April 30th.—Discharged cured, the wound having closed by first intention, leaving a good lip.

*III. Excision of Elbow Joint.*

J. M., aged 26, has had trouble with his elbow-joint for three years. He first felt pain while shaking hands, since which time the pain slowly increased, and in six months thereafter an opening was made, giving exit to what he describes as a "sausage-like material." Through the opening or fistula, which remained patulous, a small amount of pus (but a few drops) was daily discharged. Carious bone was detected. He could use his arm very well until last November, at which time he had great pain, and says he "did not sleep for thirteen nights."

The pain continuing, he came to Chicago, under the care of Prof. R. N. Isham. Finding that an operation was necessary, Prof. Isham, on Feb. 15, excised the joint at the college, using the H flaps, and sawing off the entire articular facets of the bones. There was some hæmorrhage, which was readily controlled by styptics.

The patient was then sent to the hospital, and the arm dressed in a semi-flexed position. There was little swelling or pain. The patient remained in bed but two days.

March 1st.—Union by first intention, except at the points at which injections were made, which remain open.

March 15th.—Patient left the hospital to-day. The fistulæ have not entirely healed. Passive motion has been kept up, but there is very limited motion.

April 21st.—Re-entered. Elbow doing poorly; considerable discharge. Dressed antiseptically. Gave tincture of iron internally. His general health is poor.

May 15th.—The fistulæ have healed; swelling subsiding. There is now considerable motion.

June 11th.—Nearly well. Will leave in a few days. Can flex the forearm to nearly right angles with the arm. Pronation and supination are not entirely lost.

